# Toxicity Classification of Oxide Nanomaterials: Effects of Data Gap Filling and PChem Score-based Screening Approaches

**DOI:** 10.1038/s41598-018-21431-9

**Published:** 2018-02-16

**Authors:** My Kieu Ha, Tung Xuan Trinh, Jang Sik Choi, Desy Maulina, Hyung Gi Byun, Tae Hyun Yoon

**Affiliations:** 1 Department of Chemistry, College of Natural Sciences, Hanyang University, Seoul, 04763 Republic of Korea; 2 Division of Electronics, Information and Communication Engineering, Kangwon National University, Samcheok, Kangwon-do 24341 Republic of Korea

## Abstract

Development of nanotoxicity prediction models is becoming increasingly important in the risk assessment of engineered nanomaterials. However, it has significant obstacles caused by the wide heterogeneities of published literature in terms of data completeness and quality. Here, we performed a meta-analysis of 216 published articles on oxide nanoparticles using 14 attributes of physicochemical, toxicological and quantum-mechanical properties. Particularly, to improve completeness and quality of the extracted dataset, we adapted two preprocessing approaches: data gap-filling and physicochemical property based scoring. Performances of nano-SAR classification models revealed that the dataset with the highest score value resulted in the best predictivity with compromise in its applicability domain. The combination of physicochemical and toxicological attributes was proved to be more relevant to toxicity classification than quantum-mechanical attributes. Overall, by adapting these two preprocessing methods, we demonstrated that meta-analysis of nanotoxicity literatures could provide an effective alternative for the risk assessment of engineered nanomaterials.

## Introduction

Metal oxide nanoparticles (NPs) are an important sub-category of engineered nanomaterials (ENMs), as they have very wide usage such as in cosmetics, textiles, paints, water-treatment agents, solar batteries, and automobile catalytic converters^1^. However, it was also reported that metal oxide NPs may cause persistent stress to living organisms, including humans^2^. Therefore, knowledge about the relationship between the characteristics of metal oxide NPs and their toxicity becomes critical for hazard assessment of ENMs. In addition to using experimental approaches, there is a need for *in silico* methods to develop nanoscale structure-activity relationships (nano-SARs) that analyze the correlations between NPs’ properties and their toxicity endpoints. There have been several studies on nano-SAR model development for predicting the biological effects of diverse nanomaterials^3–8^. However, the data that have been used for qualitative classification or quantitative regression models were mostly generated from individual studies with small datasets rather than large datasets comprehensively collected from published literature^9^. This has driven our research toward developing a predictive model that describes the relationship between physicochemical properties and cytotoxicity of metal oxide NPs based on a comprehensive dataset gathered from the literature.

Literature data mining, or meta-analysis, has been successfully used in nanotoxicological studies^9,10^. This approach can provide a systematic comparison of the data in the literature and a critical understanding of the relationships between the physicochemical properties, experimental conditions, and bioactivity of the nanomaterials. However, there are two important issues which were not examined yet in previous publications: wide heterogeneities of published data in terms of data quality and data completeness^11^. Data quality issue concerns the lack of standardized test protocols between laboratories, while data completeness issue concerns the amount of missing data^12,13^. In this study, we proposed potential solutions to these two issues. To deal with data completeness, we adapted a data gap filling approach, which involved replacement of missing data with information from manufacturers’ specifications or other references using the same nanomaterials or with estimation from other physicochemical properties. To overcome the data quality issue, we applied a novel scoring framework to evaluate the quality of physicochemical data in terms of their source and measurement methods. Then, we evaluated the effects of the data gap filling and quality screening approaches based on the performance of the nano-SAR models.

## Results

### Data compilation

**Figure 1:**
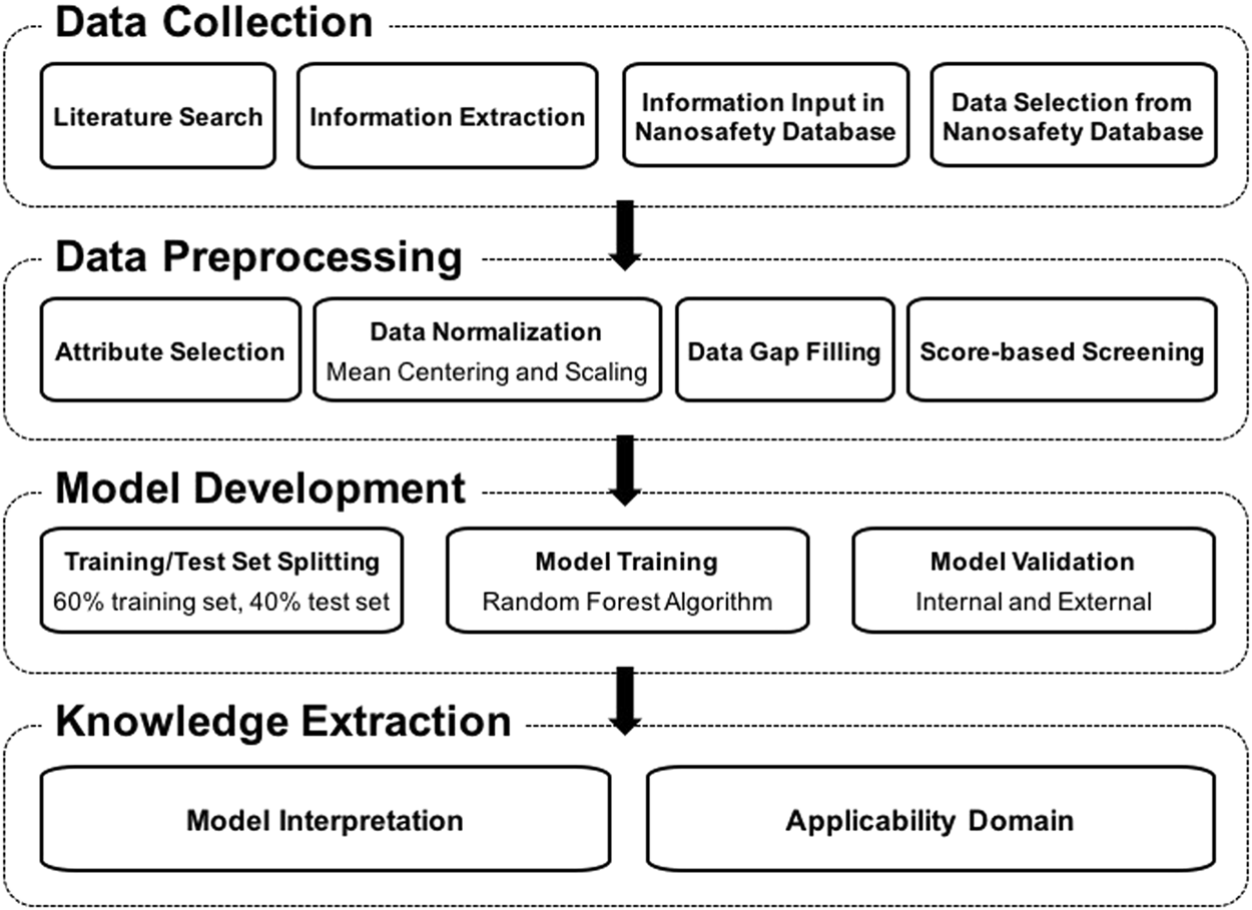
Workflow of data collection, preprocessing, model development, validation and interpretation.

Figure 1 summarizes the workflow for the meta-analysis, with details described in the Methods section. From the S2NANO database (www.s2nano.org), 6,842 data rows on 1) physicochemical (PChem) properties and their measurement methods, 2) quantum-mechanical (QM) properties and 3) *in vitro* experimental conditions and cell viability (Tox) were extracted and compiled as a dataset for 26 metal oxide NPs. An overview of the dataset is shown in Table 1 and Supplementary Table 1. PChem attributes, which were core size, hydrodynamic size, surface charge and specific surface area, were selected to be consistent with the recommendations of a comprehensive review by Puzyn *et al*.^14^. The measurement methods for PChem attributes were included only for the purpose of data scoring and screening, but were not used in the model development afterwards. The dataset also includes Tox attributes for the experimental conditions of the toxicity assessment: assay, cell name, cell species, cell origin, cell type, exposure time, and dosage. The attribute “cell name” contained more than 50 items, which was excessive and could impair the generalizability of the model^9^, so it was excluded in the model development process. Cell viability was not used as an attribute in model development, but was included among Tox attributes because it was employed to define the classification endpoint. In addition to these attributes extracted directly from the S2NANO database, QM properties (enthalpy of formation, conduction band and valence band energies, electronegativity) were included, because they were reported to have correlations with NP’s toxicity endpoints. Zhang *et al*.^15^ used electronegativity, conduction band, and valence band energies to predict oxidative stress and acute pulmonary inflammation of 24 metal oxide NPs. Similarly, Liu *et al*.^4^ developed a classification model describing the cytotoxicity of 9 metal oxide NPs based on the atomization energy of the metal oxides. In our dataset, a specific NP can be exposed to several cell lines, at several concentrations, for different durations and tested by various assays. That is why one NP occupied more than one data rows, in which the PChem and QM attributes were the same but Tox attributes described different cell lines, assays, dose, time and viability percent.

**Table 1:** Data attributes.

Dosage	PChem attributes	QM attributes		Tox attributes	
Dose (μg/mL)	Core size (nm)	Surface charge (mV)	Formation enthalpy ΔH_sf_ (eV)	Assay	Cell type (normal/cancer)
	Method for core size	Method for surface charge	Conduction band energy E_c_ (eV)	Cell name	Exposure time (hours)
	Hydrodynamic size (nm)	Specific surface area (m^2^/g)	Valence band energy E_v_ (eV)	Cell species	Viability (%)
	Method for hydrodynamic size	Method for specific surface area	Electronegativity χ_MeO_ (eV)	Cell origin	

As the first OECD principle of (Q)SAR validation suggests a defined endpoint^16^, the “Toxic” and “Nontoxic” classification endpoints in this study were clearly defined. A data row was labeled “Toxic” if the viability percent was less than 50%; otherwise, it was considered “Nontoxic.” Unlike the class assignment in Liu *et al*.^4,5^ which was NP-based, this class assignment took into account the exposure condition. This would make the interpretation more explicit, as to whether a NP with a specific set of PChem and QM properties is toxic or nontoxic in a particular exposure condition.

### Effects of data gap filling and data quality screening on model performance

**Figure 2:**
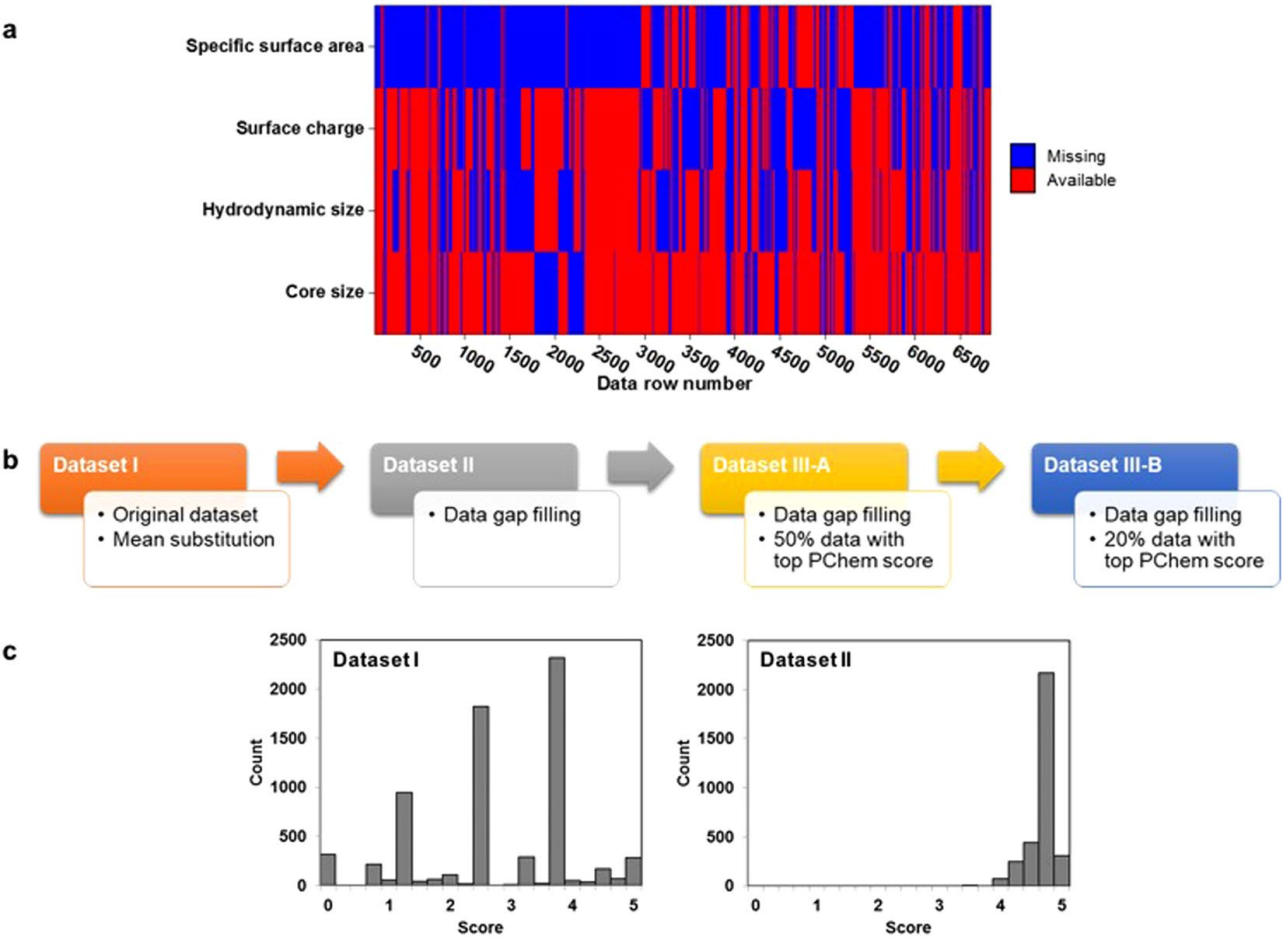
(**a**) Missing data map for PChem attributes in the original dataset; (**b**) Datasets with different preprocessing steps; (**c**) Effect of data gap filling on PChem score distribution.

The heterogeneity of the literature data led to many missing values and different quality levels. As shown in Fig. 2a, the original dataset had a great proportion of missing values in PChem attributes: 18% of core size data, 39% of hydrodynamic size data, 41% of surface charge data and 74% of specific surface area data. To overcome this problem, we proposed an approach for data gap filling (see Methods). Because we purposely selected the publications that provide the nanotoxicity data, there were no missing values and no need of data gap filling in the Tox attributes or the endpoint. There were only missing values in PChem attributes because some publications do not report on the characterization of NPs. Based on the previously reported criteria by Klimisch *et al*.^17^ and Lubinski *et al*.^13^, we have developed our own scoring criteria considering the PChem properties (i.e., PChem score) to cope with different data quality levels. We expanded the previous works by using the datasets screened by the PChem score to develop nano-SAR models in order to examine the effect of data quality on model performance. The criteria used to calculate PChem score are depicted in Table 2 and details on the scoring procedure are described in Methods. With this set of criteria, the maximum score was 5 and the minimum was 0.

**Table 2:** Scoring rules for PChem data.

Attribute	Criteria		Score
Core size	Data source	- Experimentally measured by the authors	3
		- Adapted from manufacturers’ specifications	2
		- Adapted from other references using the same nanomaterials and experimental conditions	1
		- No data	0
	Data method	- TEM	2
		- Estimated from specific surface area - Other methods (e.g., SEM/AFM)	1
		- No information	0
Hydrodynamic size	Data source	- Experimentally measured by the authors	3
		- Adapted from manufacturers’ specifications	2
		- Adapted from other references using the same nanomaterials and experimental conditions	1
		- No data	0
	Data method	- DLS/NTA	2
		- Other methods	1
		- No information	0
Surface charge	Data source	- Experimentally measured by the authors	3
		- Adapted from manufacturers’ specifications	2
		- Adapted from other references using the same nanomaterials and experimental conditions	1
		- No data	0
	Data method	- Zeta potential	2
		- Other methods	1
		- No information	0
Specific surface area	Data source	- Experimentally measured by the authors	3
		- Adapted from manufacturers’ specifications	2
		- Adapted from other references using the same nanomaterials and experimental conditions	1
		- No data	0
	Data method	- BET	2
		- Estimated from core size - Other methods	1
		- No information	0

TEM: Transmission Electron Microscopy; SEM: Scanning Electron Microscopy; AFM: Atomic Force Microscopy; XRD: X-Ray Diffraction; DLS: Dynamic Light Scattering; NTA: Nanoparticle Tracking Analysis; BET: Brunauer-Emmett-Teller method.

We divided the original dataset into several sub-datasets depending on their data gap filling approach and PChem scores (Fig. 2b). The original dataset contained missing values and was denoted as “Dataset I”. Because missing values were not allowed and must be imputed during the model development procedure, mean substitution approach was applied for the missing values in Dataset I. However, the PChem scores of the imputed data remained the same, since mean substitution was performed in the model development procedure, which was after the scoring step had been finished, as shown in Fig. 1. The missing values were then treated with the gap filling approach using the manufacturers’ specifications and/or estimations, and the resultant dataset was denoted as “Dataset II”. In Dataset II, the PChem scores were changed in accordance with data gap filling. Then, the dataset was screened based on PChem score: the top 50% and 20% of data in the descending order of PChem scores were denoted as III-A and III-B, respectively. On the basis of these datasets, nano-SAR models were developed using random forest algorithm and their performances were compared in order to investigate the effects of data preprocessing methods on the quality of nano-SAR models.

PChem score distribution of the original dataset and the dataset treated with the data gap filling approach is presented in Fig. 2c. In the original dataset (dataset I), the majority of the scores was distributed from 1 to 4. Replacing the missing values with manufacturers’ specifications and/or estimations shifted the distribution significantly to the high-score region from 4 to 5 (dataset II). This indicated that some samples initially having missing values were assigned low scores, but their scores dramatically improved when the missing values were filled in via the data gap filling approach. The replacement step not only reduced the number of missing values, but also shifted the distribution of PChem scores to a higher level and increased the amount of usable data for nano-SAR model development.

The model performance measures (i.e., sensitivity, precision, accuracy and F1 score) of five replications in external validation were averaged for each dataset and given in Table 3, while performance measures in cross-validation are presented in Supplementary Table 2. From dataset I to II, to III-A and to III-B, all performance measures constantly increased. The increase from dataset I to II confirmed that the data gap filling approach that we proposed was effective in replacing missing values and improving model predictivity. The data gap filling approach replaced the missing values in a systematic manner and thereby increased the data quality and also the amount of usable data for nano-SAR modelling. This data gap filling method would be especially appropriate for intrinsic properties such as core size and specific surface area, since these properties are independent of experimental conditions. The increase from dataset II (with no screening) to III-A (with the top 50% data of high PChem score) and then to III-B (with the top 20% data of high PChem score) indicated that datasets having higher scores would produce models with better predictivity. The PChem score was able to filter the high quality data to enhance model performance.

**Table 3:** Validation results of models built upon datasets with different preprocessing steps.

	I	II	III-A	III-B
Precision*	80%	83%	84%	91%
Sensitivity*	3%	65%	74%	88%
Accuracy	85%	94%	95%	95%
F1 score	6%	73%	79%	89%

*Precision and sensitivity were calculated with “Toxic” class as positive.

**Figure 3:**
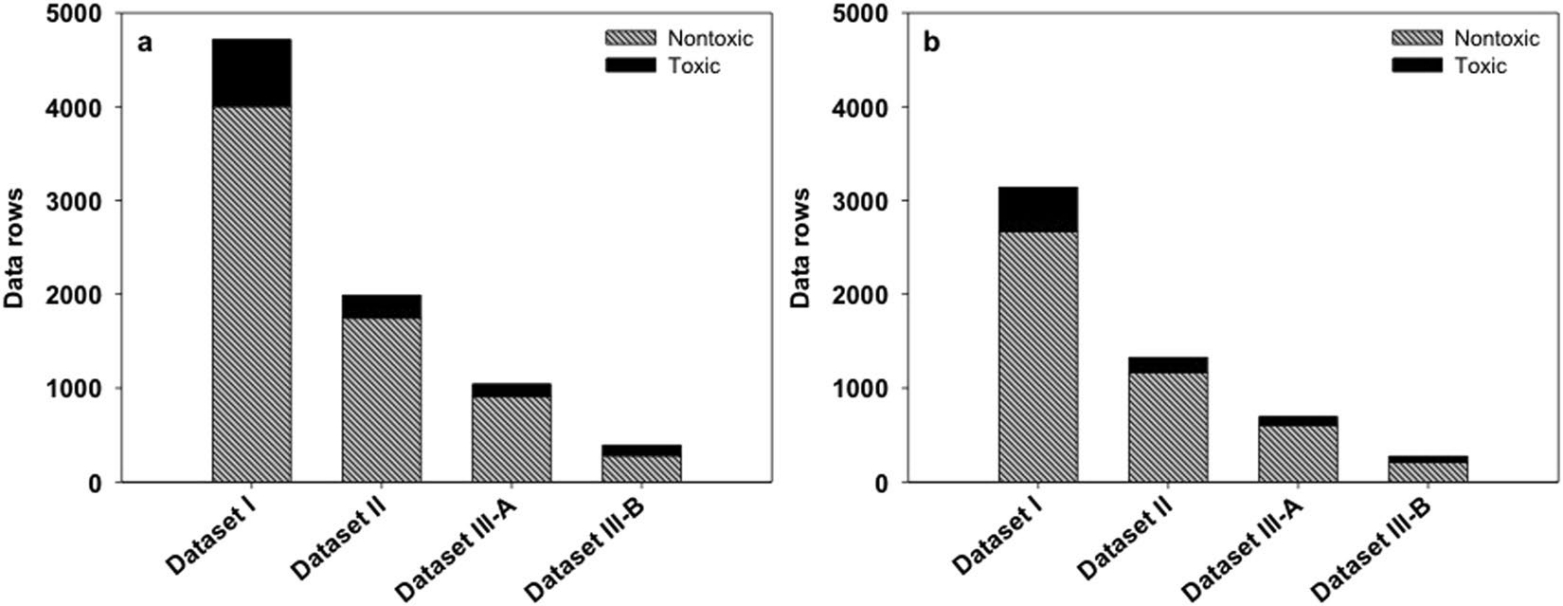
Comparison between “Toxic” and “Nontoxic” data rows in the (**a**) training set and (**b**) test set of each dataset.

Although the performance measures were high, the predictions were not balanced between Toxic and Nontoxic classes, especially in dataset I where sensitivity, which indicated the rate of true Toxic prediction, was very modest at 3%. This was caused by the imbalance between the Toxic and Nontoxic classes in the dataset. This imbalance could impair the generalization of the models, making the predictions biased to the dominant class. Figure 3 demonstrated the number of data rows in Toxic and Nontoxic classes in each dataset. It can be seen that all four datasets suffered from the imbalance problem where the majority of the data rows belonged to the Nontoxic class. In this situation, the F1 score was more suitable to evaluate the model performance than the accuracy. As expected, the F1 score for dataset I was very low, only 6%. Although it increased for datasets II, III-A and III-B, its maximum was only 89%. In order to improve the model performance, we attempted to handle this data imbalance problem by an oversampling technique SMOTE and discussed it in another manuscript.

### Applicability domain

The general definition of applicability domain (AD) was suggested by Netzeva *et al*. at the 52^nd^ workshop of the European Centre for the Validation of Alternative Methods (ECVAM): “The applicability domain of a QSAR model is the response and chemical structure space in which the model makes predictions with a given reliability”^18^. Predictions of new data points that are within the AD have high reliability, but may not be as reliable if the new data are very different from the training set^19–21^. As the third OECD principle suggests a defined domain of applicability^16^, the ADs of the developed nano-SAR models in this study were analyzed by the k-nearest neighbours algorithm using the weighted Euclidean distance (see Methods). The ADs regarding the numerical attributes of the four models developed upon the four datasets are shown in Table 4 (ADs regarding the nominal attributes are shown in Supplementary Table 5). In dataset II, any data rows containing missing values that could not be filled by the data gap filling approach were removed, so the boundary of dataset II was narrower than that of dataset I. From datasets II to III-A and to III-B, the ADs generally became smaller. This was because the datasets underwent a data quality screening step, which maintained the data with high PChem scores and removed the data with low PChem scores, thus reduced the amount of data and consequently limited the ADs. This implies that in exchange for high-quality training data and accurate prediction, the AD may become narrow. In a publication by Tong *et al*.^22^, the authors reported a decrease in prediction accuracy for two models concerning estrogen receptor binding activity as the data samples strayed further from the training domain. The accuracy was reduced by more than 50% when the data samples were about 30% away from the domain. However, in some cases such as valence band energy (E_v_) and electronegativity (χ) from III-A to III-B, the domains expanded as the datasets were screened with higher scores. This was potentially caused by the data distribution. In some datasets, the data were not evenly distributed and some data points may locate far away from the majority. When we applied the k-nearest neighbours algorithm, those biased data points would be excluded and would cause the ADs to shrink.

**Table 4:** Applicability domains regarding the numerical attributes.

Attribute	I		II		III-A		III-B	
	Min.	Max.	Min.	Max.	Min.	Max.	Min.	Max.
Dose (μg/mL)	0	10000	0	167000	0	1500	0	1500
Time (h)	0	360	1	168	2	72	6	72
Core size (nm)	2.7	629	2.7	496	5	496	5.9	193
Hydro. size (nm)	8.6	6181	8.6	2300	12.5	1463	12.5	1457
Surface charge (mV)	−63.3	61.9	−63.3	61.9	−52	61.9	−47.6	42.8
Surface area (m^2^/g)	0.8	1150	5.5	576	5.5	576	6	576
ΔH_sf_ (eV)	−64.7	−1.2	−64.7	−1.2	−26.8	−1.2	−26.8	−1.6
E_c_ (eV)	−6.6	−0.1	−6.6	−0.1	−5.2	−0.1	−5.3	−0.3
E_v_ (eV)	−11.4	−5.0	−11.3	−5.0	−11.1	−5.0	−11.4	−5.0
χ (eV)	3.2	8.3	3.4	8.3	3.4	6.8	3.8	8.3

### Attribute importance

**Figure 4:**
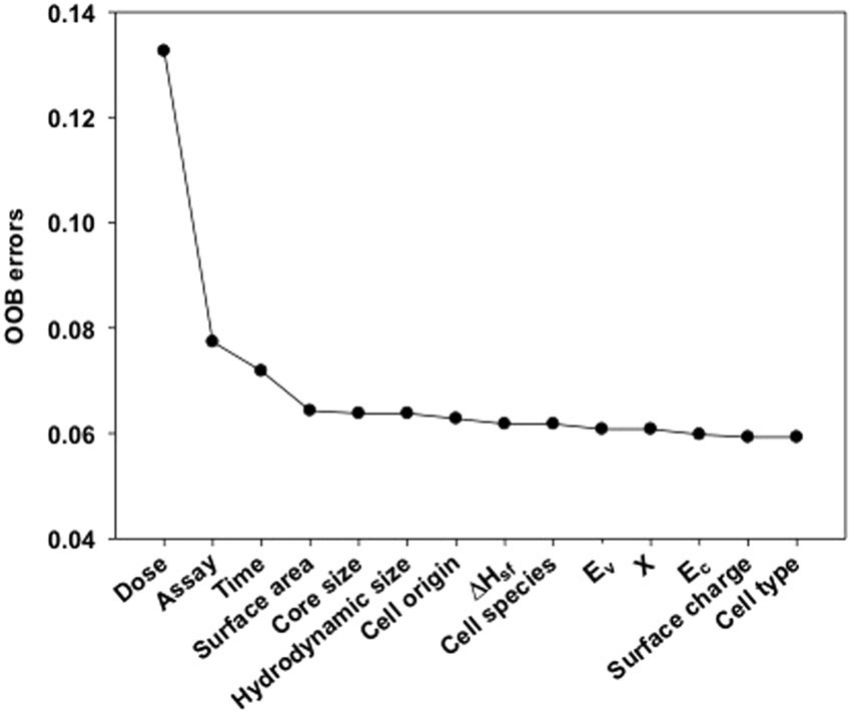
Leave-one-out OOB errors against attributes.

Table 5 shows the external validation results for the classification models developed with datasets having different combinations of attribute categories (cross-validation results are provided in Supplementary Table 3). Administered dose was used in all datasets since it is already well known to directly affect the cytotoxicity of NPs, with the highest OOB error (Fig. 4). In the case of datasets with dose and one additional attribute category (i.e., PChem, QM, or Tox attributes), addition of PChem attributes produced higher prediction accuracy and F1 score than the addition of QM or Tox attributes. After that, PChem attributes were kept in the datasets along with dose and each of the other two attribute categories were alternately added. This time, the addition of Tox attributes yielded better performance than the addition of QM attributes. These results imply that PChem attributes were the most important among these three attribute categories and they became more influential when combined with Tox attributes. This also agreed well with the results given in Fig. 4, which showed the relative importance of all attributes evaluated via comparison of the leave-one-out OOB errors. The first six attributes with the highest OOB errors were found as dose, type of assay, exposure time, surface area, core size, and hydrodynamic size. This result indicated that these attributes, which belonged to the Tox and PChem categories, were more relevant to the cytotoxicity of metal oxide NPs than the other QM attributes.

**Table 5:** Validation results of models built upon datasets with different attribute combinations.

Attributes	III-A		III-B	
	Accuracy	F1 score	Accuracy	F1 score
Dose + PChem	93%	75%	96%	92%
Dose + QM	94%	74%	92%	84%
Dose + Tox	86%	30%	89%	78%
Dose + PChem + QM	93%	75%	94%	87%
Dose + PChem + Tox	95%	81%	96%	93%

As demonstrated above, PChem attributes, together with administered dose, type of assay and exposure time had a significant influence on the cytotoxicity of NPs. Core size^23–26^, hydrodynamic size^27,28^ and surface charge^26^ of NPs have been previously reported to have critical influence on their cellular internalization process, while specific surface area is related to the reactivity of NPs with living organisms^29,30^. That is why we have chosen PChem attributes as the first example to apply both data gap filling and data quality screening approaches. However, the potentials of Tox and QM attributes also need to be addressed in future studies. In fact, Tox attributes are important in the assessment of NP toxicity as they show different physiological status and responses of cells when they are exposed to NPs^25,31,32^. QM attributes are also recognized as important factors in NP cytotoxicity as they are involved in chemical processes of NPs^3,5,33,34^. Therefore, we think that Tox and QM attributes should be considered and investigated in future studies, since they may provide further insights into the cytotoxicity of NPs, as well as improve the predictivity of nano-SAR models.

## Discussion

The data gap filling approach that we proposed was proven to improve the predictivity of the models. Substituting the missing values with manufacturers’ specifications reflected the data variance that is close to the variance of authentic data, since this approach was based on the results of quality control measurements of NPs. This approach would be especially appropriate for intrinsic properties such as core size and specific surface area, since these properties are independent of experimental conditions. It filled the data gaps in a systematic manner, thereby increased the data quality and also the amount of usable data for nano-SAR model development. Since the proposed method for data gap filling was to aid the data preparation step, it may not be directly related to the interpretation aspect of nano-SAR models. However, because data gap filling dealt with the quality and completeness issues in the curated data, it implied that we need to characterize the nanomaterials thoroughly and provide sufficient attributes concerning their structural properties, so that we can associate a specific nanomaterial identity with the observed biological activity and ensure that any mechanistic interpretation of the biological effect is reliable.

New techniques, such as read-across^35^ and interspecies^36^, might also be applicable for data gap filling. Although the meta-data analysis that we conducted in this study did not correlate the toxicity of nanomaterials on different species as in the interspecies technique, it provided a wide applicability domain covering various cell lines, cell species and assays. Furthermore, compared to the read-across technique which requires grouping the structurally similar substances, our data gap filling method was performed on a more practical and adaptable basis. In fact, considering NPs having the same product number and manufacturer as similar, and replacing the missing values with the manufacturer’s specifications or other references of the same nanomaterial, may act as a grouping strategy. This is especially helpful when the structural properties required for similarity grouping are not readily available. However, more detailed grouping strategies need to be addressed in future studies, taking into account the nano-specific properties, such as size, shape, surface chemistry and agglomeration status.

The proposed scoring framework helped us to evaluate and screen the quality of the PChem data. One might argue that by adding arbitrary values to the data gaps, the score was increased irrespectively of whether the added values were correct or incorrect. This is the reason why we proposed a scoring framework that had several levels. Regarding the score for data source, we considered the non-missing values more useful for model development than the missing values. Therefore, the values that were artificially filled in by the proposed gap filling approach were placed at the intermediate levels and assigned moderate scores (e.g., 1 or 2), higher than the “No data” and “No information” levels. Moreover, the data gaps were not filled in with irrelevant values, but values that come from the quality control measurements of the manufacturers, so the reliability should be acceptable. It is still obvious that inputting values from external sources might pose adverse effects on the model prediction. For example, inputting the hydrodynamic size from the manufacturer’s specification can be inaccurate due to differences in the dispersing medium and dispersing procedure, because the hydrodynamic size greatly depends on these factors. Thus, the data that were originally provided from experiments had the highest score because these data were the most relevant to the toxicity endpoint. Regarding the score for data method, we believed that the data would be more reproducible and reliable if they were generated by widely recognized and acknowledged techniques, such as TEM technique for measuring core size, DLS for hydrodynamic size and BET for specific surface area as suggested by the OECD^37^. That is why these data were given higher score than the data generated by less common techniques, estimated or referred to from other sources. The PChem score-based data quality screening approach also enhanced the performance of nano-SAR models. The purpose of this scoring framework is to give us flexibility in choosing suitable data for nano-SAR model development. The increased model performance of the data with high PChem score shows that data quality is crucial for the successful development of predictive and informative nano-SAR models.

The applicability domain (AD) of a predictive model is the boundary within which the model can make predictions with a given reliability. Predictions using data points outside the AD have lower confidence and accuracy than predictions using data points within the AD^16,19–21^. Therefore, AD is a very critical factor in applying the model to future data. The range-based k-nearest neighbours algorithm using weighted Euclidean distance was applied in this study to define the ADs. This method was chosen because it is simple and straightforward. It provides direct ranges of each attribute for future data analysis without requiring any additional data transformation. The broader the AD of a model, the more applicable that model is. However, the publication by Weida Tong *et al*.^22^ as well as our own results showed that as a model is widely applicable, its predictivity will be reduced. This implies that there is a compromise between how accurately a model can predict and how widely it can be applied. The specific application of the model will help determine which aspect should be the priority. Furthermore, data distribution may influence the width of ADs, especially when the ADs are analyzed via range-based or distance-based approaches, since such approaches depend greatly on data uniformity.

The model preference for PChem properties and their combination with biological parameters suggests that not all attributes are equally relevant to the cytotoxicity of metal oxide NPs. PChem attributes, together with exposure dose and time, have been reported to have an influence on the cytotoxicity of NPs. Core size can affect the cellular uptake pathway and thus influences cytotoxicity^23–26^. Hydrodynamic size provides information regarding agglomeration status of NPs, which has been reported to induce inflammatory lung injury in humans^27,28^. Surface charge, or zeta potential, indicates the surface electrostatic status of NPs. This parameter is critical in cellular internalization, as it contributes to the NP’s biocompatibility and therefore cytotoxicity^26^. Surface area represents the contact area with the biological environment and chemical reactivity, which has certain impacts on the NP’s toxicity^29,30^.

The importance of Tox attributes reflected the discussion concerning which cell lines and assays to use in ENM toxicity studies^25,32^. These factors manifest as different anatomical/biological responses of cells when they are exposed to NPs. In fact, a variety of assays and cell lines is important in the assessment of NP toxicity, as they show different cellular physiological statuses^31^. On the other hand, some viability assays, especially the colorimetric ones such as MTT or WST, were reported to show artifacts in analyzing the cytotoxicity of NPs, because the light absorbing and scattering properties of NPs may interfere with the colorimetric detection^38^. Therefore, it is necessary to make a scoring framework that takes into account the *in vitro* toxicity parameters, which concern the experimental conditions of the biological assays.

QM properties are also important factors in NP cytotoxicity. The enthalpy of formation corresponds to the energy associated with a single metal-oxygen bond in the oxides as well as the number of electrons involved in the formation reaction. It is related to the detachment of metal cations from the surface of metal oxide NPs. Additionally, Burello and Worth suggested a theoretical framework explaining that oxide NPs that possess band energy levels comparable to the cellular redox potential can participate in radical-forming reactions, leading to the generation of reactive species and the depletion of cellular antioxidants, and thus are harmful to cells^33^. However, because QM attributes are exclusive to the particle composition, they would be more effective when the modelling task is targeted for different NPs, each one having a specific size such as in Puzyn *et al*.^3^, Gajewicz *et al*.^34^, and Liu *et al*.^5^, rather than for NPs having several sizes as in this study.

## Conclusion

In this study, a comprehensive meta-analysis of published data on the cytotoxicity of metal oxide NPs was conducted. 216 publications were mined to generate 6,842 data rows with 14 attributes of physicochemical, toxicological and quantum-mechanical properties, which combined information across many individual studies. We proposed a novel data gap filling approach and a scoring framework to overcome two important challenges in computational nanotoxicology, data completeness and data quality. Our results revealed that these data gap filling and score-based quality screening approaches were effective in the classification accuracy of the nano-SAR models. By applying these approaches, we could improve the quality and completeness of the nanotoxicity data currently available in literature. Since these methods are still in an early development stage, we only demonstrated their effectiveness using qualitative classification models. However, further development and validation with regression models can be performed to investigate how data gap filling and data quality scoring can improve model prediction on a quantitative basis. Furthermore, although we focused on the PChem attributes for data gap filling and quality screening in this study, consideration on the biological parameters is in progress and may correlate the cytotoxicity of NPs with the diversity of cell lines, species and assays.

## Methods

### Meta-analysis workflow

The present study followed the workflow depicted in Fig. 1. Data were collected from published articles on cytotoxicity of metal oxide NPs. For numerical attributes, including exposure dose and time, data were normalized via mean centering and scaling. Then, missing data were filled in using replacement methods and data quality was analyzed using PChem scoring criteria. The dataset was then split into two parts: the training set (60%) and the test set (40%). The training set was used to develop classification models using random forest algorithm and to perform cross-validation, while the test set was used for external validation. Additionally, the applicability domain and attribute importance were analyzed to investigate the decision boundary of the models and the contribution of each attribute in model predictions.

### Data collection: Attributes and Endpoints

The data that were used in this study were extracted from the S2NANO database (www.s2nano.org), which collected toxicological data of various nanomaterials from several publications. From the S2NANO database, we initially identified around 600 documents related to the toxicity of metal oxide NPs. However, since many of these articles lacked information on the attributes we needed, a selection step was carried out to narrow the collection down to 216 documents. Then, 6,842 data rows were extracted and compiled as a dataset for 26 metal oxide NPs. As displayed in Table 1, these collected data included physicochemical (PChem) and quantum-mechanical (QM) properties of the metal oxide NPs along with their biological profiles (Tox). The endpoint in this study was a classification between “Toxic” and “Nontoxic”: a data row was labeled “Toxic” if the viability percent was less than 50%; otherwise, it was considered “Nontoxic”.

### Data gap filling

We proposed a novel data gap filling approach to fill in the missing values. In this approach, we filled in the missing PChem data of the target NPs with the values of the source NPs, which theoretically had similar properties to the target NPs. In the case of missing values for well-characterized commercial materials (e.g., Aeroxide P25 of Degussa-Evonik), they were replaced with the manufacturers’ characterization data of NPs that had the same brand and product number as the NPs of interest, assuming that the same NP products from the same manufacturer have similar properties. Missing values in the specific surface area could also be replaced with estimations from core size and vice versa, using equation (1):

1 $$\mathrm{SSA}=\frac{6}{{\rm{d}}\times {\rm{\rho }}}$$

where SSA is the specific surface area, d is the diameter, and ρ is the density of the NP. Missing values in QM properties were replaced with data from publications by Zhang *et al*.^15^, Gajewicz *et al*.^34^, and Liu *et al*.^5^. Since size-dependent property changes for NPs are commonly observed at sizes below 5 nm and changes for sizes above 15 nm can be neglected^39^, data regarding the QM properties from the aforementioned publications were applied to the remaining data samples of the corresponding NPs. If a data sample contained missing values that could not be replaced by this replacement approach, it would be excluded from the dataset.

Because we used nanotoxicity data with complete information on the biological parameters, such as cell viability, cell line and assay, data gap filling for the Tox attributes or the endpoint was not necessary.

### Data scoring criteria

The criteria used to calculate PChem score are depicted in Table 2. The score was calculated for PChem attributes (i.e., core size, hydrodynamic size, surface charge, and specific surface area). For each parameter, the criteria were divided into data source and data method. The data source score evaluated where the data were collected. Specifically, if the data came from experiments that were conducted and reported in an article, a data source score of 3 was assigned; if the data came from manufacturers’ specifications, a score of 2 was assigned; if the data were reused in reference to other articles, the score would be 1; and if there were no data available, the score was 0. On the other hand, the data method score describes how the data were generated. If the method is widely recognized and acknowledged (e.g., TEM/SEM/AFM for core size, DLS for hydrodynamic size, zeta potential measurement for surface charge, and BET for specific surface area), a method score of 2 was assigned; if the data were generated by less common methods or by estimation from other parameters, a score of 1 was assigned; and if no information could be provided, the score was 0. The score for each PChem attribute of one data sample was the sum of the data source and data method scores. For instance, if an article provided core size data generated by TEM, then the data sample from that article would get a score of 5 for core size. The final PChem score for a data sample was the average of the scores of all four attributes. With this set of criteria, the maximum score was 5 and the minimum was 0.

### Model development and validation

Random forest algorithm was applied for model development and validation in this study. Random forest is a machine learning algorithm that is based on a combination of tree predictors. The individual decision trees are generated using a random selection of attributes at each node to determine the split. During classification, each tree returns an independent output, and the final class is decided based on either the most voted class or the weighted integration of each tree’s result. Random forest algorithms have been shown to be appropriate for the robust meta-analysis of highly complex and heterogeneous literature data^10,40^. In a random forest, a bootstrap sample (a sample drawn with replacement) is drawn from the original data and is used to build a decision tree, with a random subset of attributes selected for each tree split. The process is replicated a prescribed number of times or until the prediction is within a target tolerance^41,42^.

In this study, the open source statistical software R (version 3.3.1) and the Rstudio integrated environment (version 1.0.136) were used. Random forest classification models were developed with the R package ‘randomForest’^42^. A fixed random state was used in all calculations to ensure that the model predictions were reproducible; in R, this was done by setting the random seeds. The models were developed based on a training set, which consisted of 60% of the dataset. The remaining 40% of the dataset made up the test set. The splitting between the training and test sets was randomized and replicated five times. For each replication, cross-validation was performed on the training set, while external validation was performed on the test set, and the validation results of five replications were averaged.

### Applicability domain

In this study, the ADs of the developed nano-SAR models were analyzed by the k-nearest neighbours algorithm using the weighted Euclidean distance. A cutoff value, D_c_, that defined a distance threshold was calculated as in equation (2)^43^

2 $${{\rm{D}}}_{{\rm{c}}}=\bar{{\rm{D}}}+{\rm{Z}}\times {\rm{s}}$$

Here, $\bar{{\rm{D}}}$ is the average, s is the standard deviation of the distances from each data sample to the other data samples in the training set, and Z is a parameter used to manipulate the confidence level. A new data sample having smaller distances to other data samples than this threshold would be considered similar to the training data at a certain confidence level and could be reliably predicted by the proposed model. We chose a Z value of 1.645, which corresponds to a confidence level of 95% in this one-tailed test. Subsets of the training data that had smaller distances than the threshold were extracted and the attribute ranges were analyzed based on these subsets. The distance threshold could therefore determine the attribute ranges within which new data samples would be similar to the training data and have reliable predictions.

### Attribute importance

The importance of the attributes was assessed by comparing the leave-one-out out-of-bag (OOB) errors. Each attribute was alternately removed from the datasets, random forest models were developed based on the datasets with the remaining attributes, and OOB errors of the models were recorded; the premise was that the exclusion of an important attribute would lead to a high OOB error. However, this approach may not account for possible dependence and complementary information provided by multiple attributes in the composite datasets, so attribute importance was additionally assessed based on the attribute categories, as in Table 1. Dose was the initially chosen attribute, as it is directly related to the cytotoxicity of the NPs. The datasets were trimmed by removing all other attributes, leaving only dose, and then expanded by sequentially adding different combinations of PChem, QM, and Tox attributes. For each combination, a random forest model was developed and the importance of the attribute categories was examined based on the model predictivity.

### Data availability

The datasets generated and/or analysed during the current study are available from the corresponding author on reasonable request.

## Electronic supplementary material

Supplementary information
