# Supplementary material for: Toxicity Classification of Oxide Nanomaterials: Effects of Data Gap Filling and PChem Score-based Screening Approaches
Source: Sci Rep. 2018 Feb 16;8:3141. doi: 10.1038/s41598-018-21431-9 (PMC5816655; doi:10.1038/s41598-018-21431-9)
Supplement: Supplementary file 1 — Supplementary information [file 41598_2018_21431_MOESM1_ESM.docx]

**Toxicity Classification of Oxide Nanomaterials: Effects of Data Gap Filling and PChem Score-based Screening Approaches**

**My Kieu Ha^1^, Tung Xuan Trinh^1^, Jang Sik Choi^2^, Desy Maulina^1^, Hyung Gi Byun^2^ and Tae Hyun Yoon^1^***

^1^Department of Chemistry, College of Natural Sciences, Hanyang University, Seoul 04763, Republic of Korea

^2^Division of Electronics, Information and Communication Engineering, Kangwon National University (Samcheok), Kangwon-do 24341, Republic of Korea

*Corresponding author: taeyoon@hanyang.ac.kr

SUPPLEMENTARY METHODS

Paper retrieval

Literature search was undertaken using many academic search engines such as Scopus (www.scopus.com), PubMed (www.ncbi.nlm.nih.gov/pubmed), Google Scholar (www.scholar.google.com), Web of Knowledge (www.thomsonreuters.com) and SciFinder (www.scifinder.cas.org). This process resulted in approximately 500 initial publications. It was also critical to obtain any available supplementary information with each publication since those, in many cases, contained important details regarding the metal oxide NPs’ properties, the toxicity assay methodology, as well as toxicity data.

Data examination

The obtained publications underwent an examination to assess if the necessary data for all the attributes chosen for this study were provided and could be possibly extracted. Publications that only dealt with toxicity tests without focus on NP characterization were excluded, since they could not provide information on the attributes in nano-SAR modelling in this study. In many cases, key information was not directly stated in the articles but rather referred to in a cited reference. Those cases required secondary reference search to locate and extract the needed parameters.

Data extraction

The data were extracted manually and then input in the S2NANO database ([www.s2nano.org](http://www.s2nano.org)). The S2NANO database is divided into 4 sub-databases (DB), which are Information DB, Material DB, PChem DB and Toxicity DB, in hierarchical order. Information DB contains categories regarding the identification of articles, such as ‘Journal’, ‘Year’ and ‘Authors’. Each article is encoded by the PubMed ID number given by the US National Library of Medicine, National Institutes of Health. Material DB contains categories concerning the nanoparticles used in the publications in Information DB, such as ‘Material type’, ‘Manufacturer’ and ‘Manufacturing method’. PChem DB stores information about physico-chemical properties of the nanoparticles such as shape, core size, hydrodynamic size, surface charge, specific surface area and dissolution. Toxicity DB stores nanoparticles’ toxicity data, which are divided into *in vitro*, *in vivo* and *eco* categories. For example, in the *in vitro* category, there are data regarding the cell line that is used for testing, viability, LC_50_ and generation of reactive oxygen species. In many cases, the toxicity data were not presented in a tabular format but rather as dose-response curves or bars. The values of the data points on these plots were retrieved manually using ImageJ software (ImageJ 1.41n, NIH, USA) with a self-written macro for plot analysis. The source species, the tissue of origin and the type (normal/cancer) of the cell lines also needed to be clarified. For this step, the American Type Culture Collection website (www.atcc.org) was used to get the necessary information.

The S2NANO database covers many types of nanomaterials (e.g., metals, metal oxides, carbon nanotubes, quantum dots) as well as *in vitro*, *in vivo* and *eco* toxicological data. However, for the scope of this study, we only extracted the data concerning metal oxide NPs and *in vitro* toxicity.

SUPPLEMENTARY FIGURES


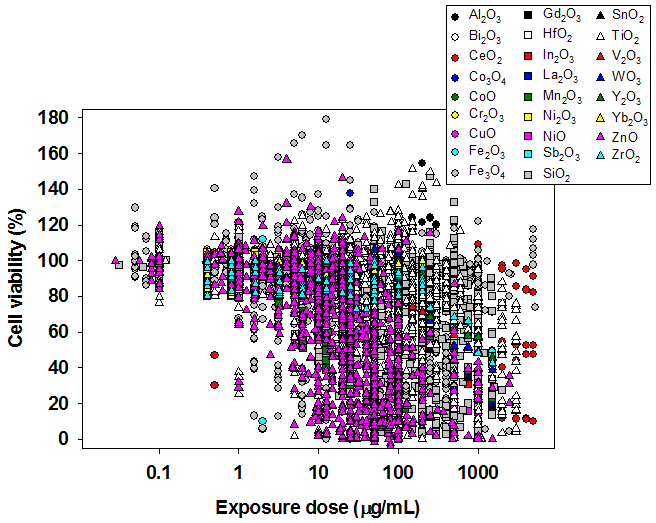


**Supplementary Figure 1.** Plot of cell viability against exposure dose by material type for most of the initial dataset. A few data points outside of the ranges shown have been omitted for clarity of presentation.


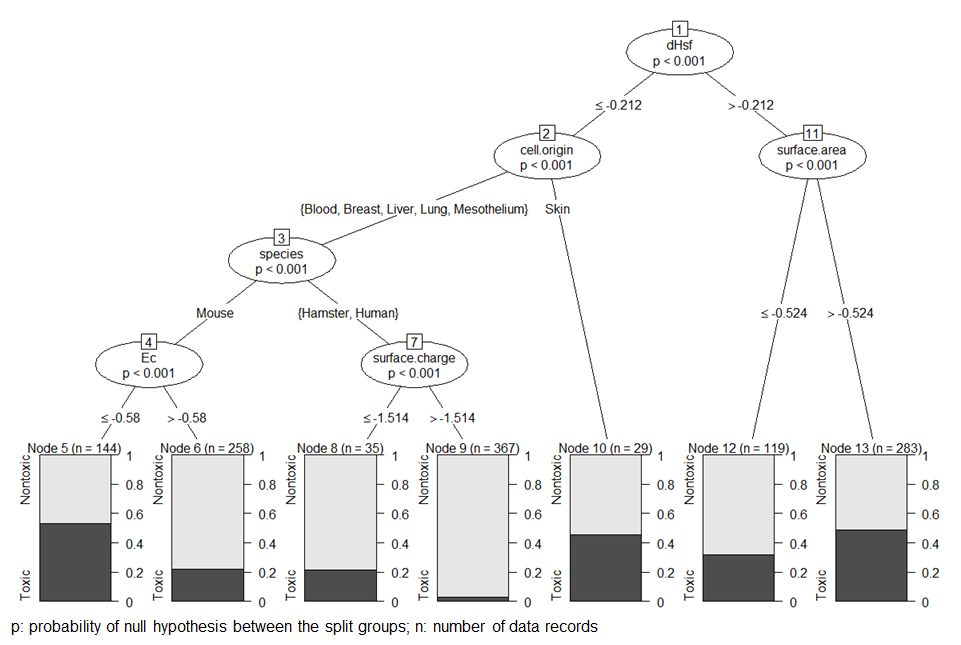


**Supplementary Figure 2.** A sample tree from random forest model. The tree is read from the top down, following each branch to its terminal leaf. In this tree, the terminal leaf does not specifically point out which class the observation belongs to. Instead, it provides the probability of the observation belonging to Toxic or Nontoxic class. For example, if the normalized formation enthalpy (ΔH_sf_) is larger than ‑0.212 eV and normalized specific surface area is larger than ‑0.524 m^2^/g, then there is only 50% percent chance that the observation is Nontoxic. However, if normalized ΔH_sf_ is equal to or smaller than ‑0.212, cell origin is one of blood, breast, liver, lung or mesothelium, cell species is either hamster or human and normalized surface charge is larger than ‑1.514, then the observation has above 90% chance of being Nontoxic. 500 trees in a random forest model were developed in a similar manner and provided class probability for each new observation.

SUPPLEMENTARY TABLES

Supplementary Table 1. Overview of data attributes

| **1. Material** | **26** |  | **4. Biological attributes** |  |  |  |  |
| --- | --- | --- | --- | --- | --- | --- | --- |
| Fe_3_O_4_ | 23% |  | ***4.1 Assay*** | ***31*** |  | ***4.3 Cell origin*** | ***32*** |
| TiO_2_ | 22% |  | MTT | 36% |  | Lung | 29% |
| ZnO | 18% |  | MTS | 13% |  | Blood | 21% |
| SiO_2_ | 13% |  | LDH | 12% |  | Bone | 8.1% |
| Others | 24% |  | ATP | 6.9% |  | Skin | 6.4% |
|  |  |  | WST | 6.7% |  | Liver | 5.6% |
| **2. PChem attributes** |  |  | Trypan blue | 5.5% |  | Brain | 4.1% |
| ***2.1 Core size*** |  |  | Alamar blue | 3.5% |  | Embryo | 3.2% |
| 2.72 to 629 nm |  |  | NRU | 2.8% |  | Fibroblast | 3.0% |
| ***2.2 Hydrodynamic size*** |  |  | CCK-8 | 2.5% |  | Breast | 2.9% |
| 8.6 to 6181 nm |  |  | CytoTox-Glo | 2.3% |  | Colon | 2.8% |
| ***2.3 Surface charge*** |  |  | CellTiter-Glo | 1.3% |  | Somatic cell hybrid | 2.6% |
| -63.3 to 61.9 mV |  |  | CellTiter-Blue | 1.0% |  | Cervix | 2.0% |
| ***2.4 Specific surface area*** |  |  | Calcein AM | 0.8% |  | Umbilical vein | 1.6% |
| 1.8 to 1150 m^2^/g |  |  | FDA uptake | 0.8% |  | Others | 8.2% |
|  |  |  | Others | 5.3% |  |  |  |
| **3. Fundamental attributes** |  |  |  |  |  | ***4.4 Cell type*** | ***2*** |
| ***3.1 ΔH_sf_*** |  |  | ***4.2 Cell species*** | ***10*** |  | Cancer | 53% |
| -64.7 to -1.2 eV |  |  | Human | 68% |  | Normal | 47% |
| ***3.2 E_c_*** |  |  | Mouse | 29% |  |  |  |
| -6.6 to -0.1 eV |  |  | Hamster | 1.1% |  | ***4.5 Exposure time*** |  |
| ***3.3 E_v_*** |  |  | Fish | 0.4% |  | 0 to 360 h |  |
| -11.4 to -5.0 eV |  |  | Dog | 0.3% |  |  |  |
| ***3.4 χ_MeO_*** |  |  | Monkey | 0.2% |  | **5. Exposure dose** |  |
| 3.2 to 8.3 eV |  |  | Others | 0.4% |  | 0 to 667000 μg/mL |  |

Supplementary Table 2. Five replicate results of cross-validation for models built upon datasets with different preprocessing steps

Replication 1

|  | **I** | **II** | **III-A** | **III-B** |
| --- | --- | --- | --- | --- |
| **Precision** | 96% | 87% | 83% | 89% |
| **Sensitivity** | 4% | 64% | 69% | 83% |
| **Accuracy** | 85% | 94% | 94% | 93% |
| **F1 score** | 7% | 74% | 75% | 86% |

Replication 2

|  | **I** | **II** | **III-A** | **III-B** |
| --- | --- | --- | --- | --- |
| **Precision** | 96% | 88% | 85% | 88% |
| **Sensitivity** | 3% | 65% | 70% | 84% |
| **Accuracy** | 85% | 95% | 95% | 93% |
| **F1 score** | 7% | 75% | 77% | 86% |

Replication 3

|  | **I** | **II** | **III-A** | **III-B** |
| --- | --- | --- | --- | --- |
| **Precision** | 92% | 87% | 82% | 87% |
| **Sensitivity** | 2% | 66% | 69% | 84% |
| **Accuracy** | 85% | 95% | 94% | 93% |
| **F1 score** | 3% | 75% | 75% | 85% |

Replication 4

|  | **I** | **II** | **III-A** | **III-B** |
| --- | --- | --- | --- | --- |
| **Precision** | 96% | 85% | 85% | 90% |
| **Sensitivity** | 4% | 60% | 70% | 84% |
| **Accuracy** | 85% | 94% | 95% | 93% |
| **F1 score** | 7% | 71% | 77% | 87% |

Replication 5

|  | **I** | **II** | **III-A** | **III-B** |
| --- | --- | --- | --- | --- |
| **Precision** | 100% | 87% | 83% | 89% |
| **Sensitivity** | 4% | 67% | 68% | 82% |
| **Accuracy** | 85% | 95% | 94% | 93% |
| **F1 score** | 7% | 76% | 75% | 85% |

Supplementary Table 3. Cross-validation for models built upon datasets with different attribute combinations

|  | **III-A** | | **III-B** | |
| --- | --- | --- | --- | --- |
| **Attributes** | **Accuracy** | **F1 score** | **Accuracy** | **F1 score** |
| **Dose + PChem** | 93% | 71% | 93% | 87% |
| **Dose + QM** | 94% | 71% | 91% | 81% |
| **Dose + Tox** | 89% | 40% | 83% | 68% |
| **Dose + PChem +QM** | 93% | 71% | 92% | 85% |
| **Dose + PChem + Tox** | 94% | 76% | 93% | 86% |

Supplementary Table 4. Five replicate results of external validation for models built upon datasets with different preprocessing steps

Replication 1

|  | **I** | **II** | **III-A** | **III-B** |
| --- | --- | --- | --- | --- |
| **Precision** | 91% | 84% | 83% | 91% |
| **Sensitivity** | 4% | 66% | 69% | 87% |
| **Accuracy** | 86% | 94% | 94% | 95% |
| **F1 score** | 7% | 74% | 75% | 89% |

Replication 2

|  | **I** | **II** | **III-A** | **III-B** |
| --- | --- | --- | --- | --- |
| **Precision** | 87% | 84% | 81% | 91% |
| **Sensitivity** | 4% | 67% | 66% | 85% |
| **Accuracy** | 85% | 94% | 94% | 94% |
| **F1 score** | 8% | 75% | 72% | 88% |

Replication 3

|  | **I** | **II** | **III-A** | **III-B** |
| --- | --- | --- | --- | --- |
| **Precision** | 100% | 82% | 88% | 91% |
| **Sensitivity** | 2% | 67% | 67% | 87% |
| **Accuracy** | 85% | 94% | 95% | 95% |
| **F1 score** | 3% | 74% | 76% | 89% |

Replication 4

|  | **I** | **II** | **III-A** | **III-B** |
| --- | --- | --- | --- | --- |
| **Precision** | 95% | 89% | 77% | 91% |
| **Sensitivity** | 4% | 67% | 73% | 87% |
| **Accuracy** | 86% | 95% | 94% | 95% |
| **F1 score** | 8% | 77% | 75% | 89% |

Replication 5

|  | **I** | **II** | **III-A** | **III-B** |
| --- | --- | --- | --- | --- |
| **Precision** | 91% | 82% | 83% | 91% |
| **Sensitivity** | 5% | 61% | 66% | 87% |
| **Accuracy** | 86% | 94% | 94% | 95% |
| **F1 score** | 9% | 70% | 73% | 89% |

Supplementary Table 5. Applicability domain regarding the nominal attributes

| **Attribute** | **I** | **II** | **III-A** | **III-B** |
| --- | --- | --- | --- | --- |
| **Assay** | AK  Alamar blue  Annexiv V/PI staining  ATP  BrdU  Calcein AM  CCK-8  CellTiter-Blue  CellTiter-Glo  CytoTox-Glo  FDA uptake  LDH  Live/Dead viability  MTS  MTT  NRU  PicoGreen  PrestoBlue  RCC  Resazurin assay  RTCA  SRB  Tali apoptosis  TOTO-3  Tox tracker  Trypan blue  WST | Alamar blue  Annexiv V/PI staining  ATP  CCK-8  CellTiter-Blue  CellTiter-Glo  CytoTox-Glo  LDH  MTS  MTT  NRU  PicoGreen  PrestoBlue  RTCA  Trypan blue  WST | Alamar blue  Annexiv V/PI staining  ATP  CCK-8  CellTiter-Glo  CyQuant assay  CytoTox-Glo  LDH  MTS  MTT  PrestoBlue  RTCA  Trypan blue  WST | Annexiv V/PI staining  ATP  CCK-8  CellTiter-Glo  CytoTox-Glo  LDH  MTS  MTT  RTCA  WST |
| **Cell species** | Cow  Dog  Fish  Hamster  Human  Mouse | Chick  Hamster  Human  Mouse | Human  Hamster  Mouse | Human  Hamster  Mouse |

Supplementary Table 5. (continued) Applicability domain regarding the nominal attributes

| **Attribute** | **I** | **II** | **III-A** | **III-B** |
| --- | --- | --- | --- | --- |
| **Cell origin** | Adipose tissue  Adrenal gland  Bladder  Blood  Bone  Brain  Breast  Cervix  Colon  Embryo  Eye  Fibroblast  Heart  Kidney  Liver  Lung  Lymphocyte  Neck  Nose  Ovary  Skin  Somatic cell hybrid  Spleen  Stomach  Umbilical vein | Adipose tissue  Blood  Bone  Brain  Breast  Colon  Forebrain  Liver  Lung  Skin | Adipose tissue  Blood  Bone  Breast  Liver  Lung  Nose  Skin | Blood  Liver  Lung  Nose  Skin |
